# Recent Advances in Fabrication and Applications of Yttrium Aluminum Garnet-Based Optical Fiber: A Review

**DOI:** 10.3390/ma17143426

**Published:** 2024-07-11

**Authors:** Yuli Pang, Xu Lu, Xin Zhang, Ziheng Miao, Min Sun, Guowu Tang, Jialong Li, Qilai Zhao, Changsheng Yang, Dongdan Chen, Qi Qian, Zhuo Xu

**Affiliations:** 1 Laboratory of Functional Materials, School of Materials Science and Engineering, Xi’an University of Technology, Xi’an 710048, China; 2 Electronic Materials Research Laboratory, Key Laboratory of the Ministry of Education, International Center for Dielectric Research, School of Electronic and Information Engineering, Xi’an Jiaotong University, Xi’an 710049, China; 3 School of Physics and Optoelectronic Engineering, Guangdong University of Technology, Guangzhou 510006, China; 4 State Key Laboratory of Luminescent Materials and Devices, Guangdong Provincial Key Laboratory of Fiber Laser Materials and Applied Techniques, Guangdong Engineering Technology Research and Development Center of Special Optical Fiber Materials and Devices, Institute of Optical Communication Materials, South China University of Technology, Guangzhou 510640, China

**Keywords:** yttrium aluminum garnet, optical fiber, fabrication, applications

## Abstract

Yttrium aluminum garnet (YAG)-based optical fiber is one of the research hotspots in the field of fiber lasers due to its combined advantages of a wide doping range of rare earth ions and the high mechanical strength of YAG material, as well as the flexibility and small size of the fiber structure. YAG-based optical fibers and related laser devices can be used in communication, sensing, medicine, etc. A comprehensive review of YAG-based optical fibers is provided in this paper. Firstly, the fabrication processes of YAG-based optical fibers are summarized and the structure and properties of fibers are classified and compared. Secondly, according to the optical wavelength regions, rare earth-doped YAG-based optical fibers for the applications of single-frequency and mode-locked fiber lasers are summarized. Lastly, the development challenges in both the fabrication and applications of YAG-based optical fibers are discussed.

## 1. Introduction

With the rapid development of information technology, the requirements of functional fibers are constantly increasing. For traditional silica fiber, it is impossible to achieve high gain and high power output due to its low concentration of rare earth doping [1,2]. The low nonlinear optical application rate and narrow transmission window in the near-infrared region of silica fiber also limit the development of nonlinear devices and hinder diversified applications [3]. Furthermore, the thermal management capabilities and mechanical properties of silica fiber are no longer sufficient to meet the needs of extreme working environments [4]. In order to address the above shortcomings of traditional silica fibers in applications, there is an urgent need to develop new functional optical fiber materials.

Yttrium aluminum garnet (YAG) is one of the excellent hosts for laser applications due to its wide transmission range of 0.28~5.5 μm, high thermal conductivity of 13.0 Wm^−1^K^−1^, and wide doping concentration of various active ions (Ce^3+^, Yb^3+^, Er^3+^, Cr^4+^, etc.). In recent years, YAG-based fibers have been energetically developed and show great potential in the field of fiber lasers. With the advantages of a low pumping threshold, low noise, high slope efficiency, short attenuation time, and high luminous efficacy, etc., YAG-based fiber lasers have been used in a wide range of applications in military, coherent communication, atmospheric remote sensing, high-resolution spectroscopy, and biomedical fields, among others.

In this review, firstly, the fabrication methods of YAG-based fibers are introduced and their advantages and limitations are analyzed. Subsequently, the single-frequency and mode-locked characteristics of YAG fiber lasers of the wavelengths of 1.0 μm, 1.5 μm, and 2.0 μm are discussed and compared, respectively. Lastly, a comprehensive overview of the challenges in the fabrication and application of YAG-based optical fibers is provided, followed by proposing future development strategies.

## 2. Fabrication of YAG-Based Optical Fibers

**Figure 1:**
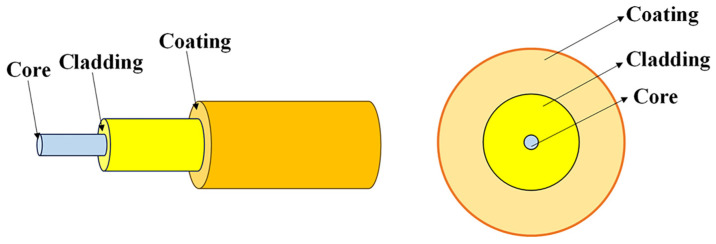
Schematic structure of optical fiber.

The common structure of optical fibers is shown in Figure 1; it is generally divided into three parts: core, cladding, and coating. A coaxial optical waveguide structure is composed of a fiber core with a higher refractive index and cladding with a lower refractive index, allowing light to propagate inside the fiber under internal total reflection conditions. The coating is used to prevent external damage and improve the mechanical strength of the optical fiber.

The common fabrication methods of functional fibers can be classified into three categories. The first is based on crystal growth methods, such as the laser-heated pedestal growth method and the micro-pulling down method, to fabricate one-dimensional crystals as the core, followed by cladding fabrication through casing or other methods. The second is by filling the core material into a capillary tube, such as in the pressure-assisted melt filling method and high-pressure chemical vapor deposition method, which are commonly utilized in the fabrication of silicon fibers [5,6,7,8]. The third is a kind of improved method of traditional fiber drawing, such as the melt-in-tube method. For the fabrication of YAG-based fibers, the laser-heated pedestal growth method, the micro-pulling down method, and the melt-in-tube method are widely used.

### 2.1. Laser-Heated Pedestal Growth Method

**Figure 2:**
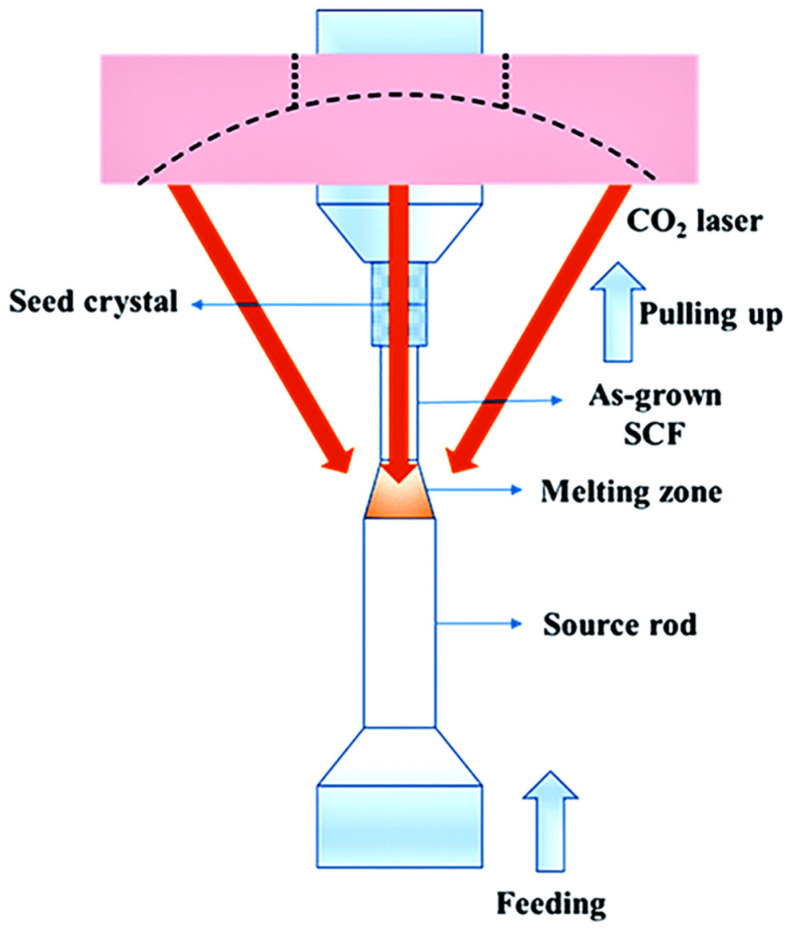
Schematic of laser-heated pedestal growth [9]. Copyright © 2019 RSC Advances.

Laser-heated pedestal growth (LHPG) is based on one-dimensional crystal growth, commonly used for the fabrication of single-crystal fibers (SCFs). The principle of this method is illustrated in Figure 2 [9]. A crystal source rod is vertically mounted on a feeding pedestal, and the tip of the rod is locally heated by a laser to form a small melt region. Subsequently, a seed crystal contacts the melt region and pulls upwards, while the source rod feeds upwards at a controlled speed for raw material compensation. Consequently, the single-crystal fibers of the one-dimensional structure are gradually grown. As early as 1975 in Bell Laboratory, Nd^3+^:YAG crystal was successfully fabricated by the LHPG method and realized laser output [10]. Subsequently, researchers from Rutgers University and Clemson University paid great attention to the fabrication of YAG single crystals based on the LHPG method. However, the direct contact between the YAG crystals and the air (without cladding) resulted in increased numerical aperture, larger mode numbers, and greater loss, which affected the quality and efficiency of the output laser. In order to solve the above problems, the fabrication of YAG crystal fibers with cladding was proposed. Furthermore, suitable cladding also acts as a protective layer on the fiber. According to the growth processes of crystal and cladding, the fabrication of YAG fibers with cladding by the LHPG method can be classified into two categories: the first is co-drawing laser-heated pedestal growth (CDLHPG) based on the simultaneous growth of the crystal core and cladding, using a source rod composed of a YAG single crystal as the core and silica as the cladding. The second is based on the separate growth of the core and cladding, which generally uses LHPG to grow a one-dimensional crystal core and then uses other methods to grow the cladding.

#### 2.1.1. Simultaneous Growth of Core and Cladding

**Figure 3:**
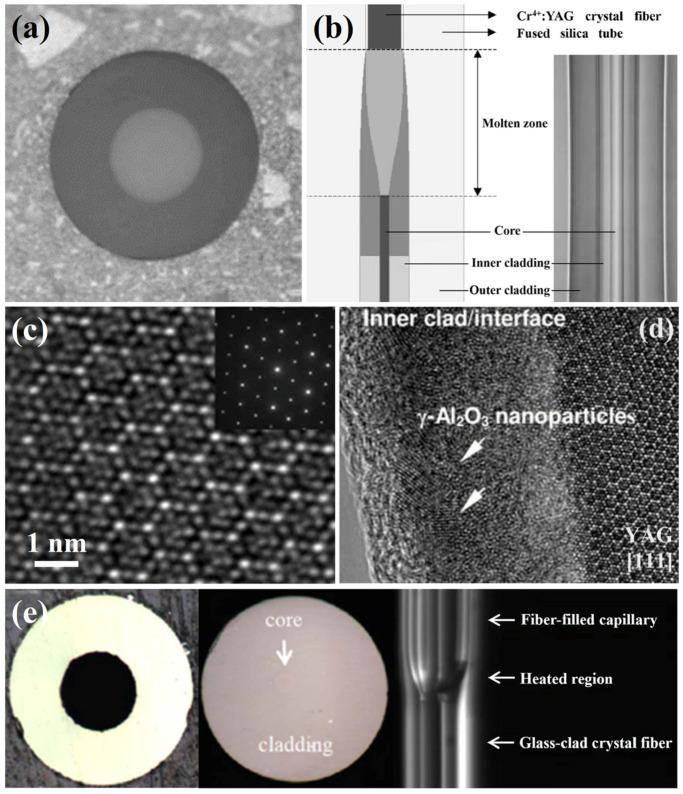
(**a**) Photograph of a silica-clad Cr:YAG fiber with a core diameter of 29 μm [11]. [Reprinted] with permission from ref. [11] © Optics Letters. (**b**) The left side of the figure shows a schematic of the fusion zone during the growth of the YAG fiber and the right side shows a side view of the grown double-clad Cr:YAG fiber [12]. [Reprinted] with permission from ref. [12] © Optics Letters. (**c**) TEM images of silica-coated Cr: YAG fiber core regions prepared by the LHPG method [13]. [Reprinted] with permission from ref. [13] © Optics Letters. (**d**) TEM images of the YAG/SiO_2_ interface region [14]. [Reprinted] with permission from ref. [14] © Optica Publishing Group. (**e**) Optical fiber microscope photographs of glass-coated crystals grown by the CDLHPG method [16]. Copyright © 2013 Optical Materials Express.

In 2004, Huang and his team at Taiwan University conducted a series of fabrication studies of YAG crystal fibers using the CDLHPG method and analyzed the diffusion between the core and shell [11,12,13,14,15,16,17]. Lo et al. fabricated Cr:YAG crystal fiber in a diameter of 29 μm with silica cladding by CDLHPG [11]. As shown in Figure 3a, the interface of the fiber was relatively good, but the content of SiO_2_ in the core was as high as 64.9 wt%. Using the Cr:YAG crystal fiber above, the amplification of spontaneous emission (ASE) at 1.2–1.55 μm was successfully achieved. Subsequently, his team fabricated a double-clad Cr:YAG crystal fiber to reduce the SiO_2_ proportion [12]. The silica capillary of the outer layer and the crystal nanostructure of the inner layer are shown in Figure 3b. It was found that the fiber core contained 39.8 wt% SiO_2_, indicating that the double cladding reduced diffusion to a certain extent. In 2008, Lai et al. optimized the LHPG process parameters and fabricated Cr:YAG double-clad crystal fiber through the same heating system, further reducing the SiO_2_ concentration in the core to about 20 wt%. Moreover, the clear diffraction spot on the right side of Figure 3c proved the existence of a YAG single-crystal structure in the core [13]. In 2009, Lai conducted the same double-clad Cr:YAG fabrication experiments [14] and concluded that the interface layer between YAG/SiO_2_, as captured by TEM in Figure 3d, consisted of Cr^3+^:γ-Al_2_O_3_ nanocrystals instead of the amorphous glass previously recognized. In 2011, the same research group fabricated Ce^3+^/Sm^3+^ co-doped YAG double-clad crystal fiber [15], and the maximum concentrations of Ce^3+^ and Sm^3+^ in the core were 1.15 at% and 8.23 at%, respectively. They successfully used the Ce^3+^/Sm^3+^:YAG fiber to generate broadband high-power emission at the visible wavelength band, demonstrating the potential of this light source in optical coherence tomography applications. Then, in order to compensate for the multimode defect caused by the large refractive indices difference between the core and cladding, Hsu et al. successfully fabricated YAG crystal fibers with high-refractive-index N-SF57 glass cladding, as shown in Figure 3e [16]. The experiments proved the feasibility of using high-refractive-index glass to effectively reduce the number of guide modes. In the same year, Hsu further investigated the relationship between high-refractive-index glass and the number of guide modes. They used N-LaSF9 glass with different refractive indices as cladding and a slow cooling rate annealing process to adjust the refractive index difference between the core and cladding. Then, a YAG crystal fiber with a core diameter of 40 μm and a cladding diameter of 945 μm was fabricated. Through further optical measurement, the assumption that the CDLHPG process induces a reduction in the glass cladding refractive index was verified and less-mode optical transmission was realized [17].

#### 2.1.2. Separate Growth of Core and Cladding

In the fabrication of YAG single-crystal fiber by the CDLHPG method, molten glass under high temperatures dissolves and consumes the YAG crystal, and the mismatch of thermal conductivities between the glass and crystal leads to the occurrence of thermal gradient phenomena. In recent years, the research trend has gradually transitioned from YAG glass-clad crystal fibers to YAG fully crystalline fibers.

**Figure 4:**
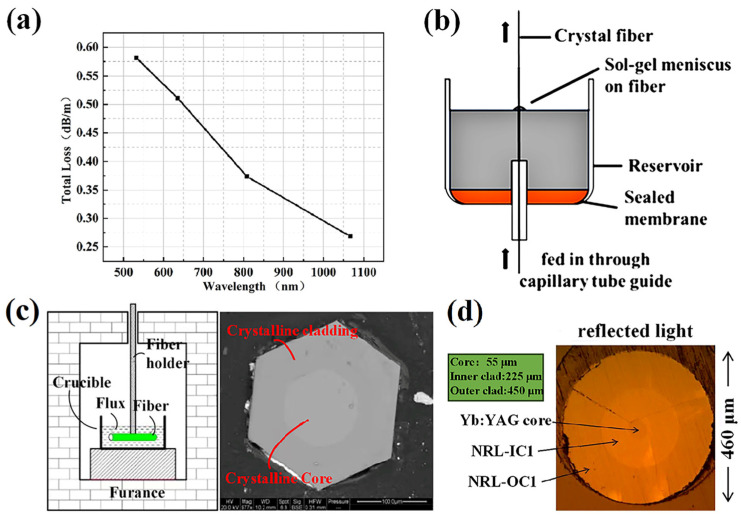
(**a**) Loss of single-crystal YAG fibers at four laser wavelengths [19]. Copyright © 2016 Optics Express. (**b**) Stretch-coating device for fine fibers [20]. Copyright © 2017 Crystals. (**c**) A simplified view of the LPE growth system used to grow the crystal cladding and SEM cross-section of a Yb^3+^:YAG holocrystalline fiber [21]. Copyright © 2018 Optics Express. (**d**) Micrograph of prepared 3% Yb^3+^:YAG end face [22]. [Reprinted] with permission from ref. [22] © SPIE.

In 2013, Nie’s team conducted a systematic study of the ion distribution and scattering loss of YAG fibers [18]. Er^3+^-doped YAG single-crystal fibers were grown by the LHPG method, and the radial distribution of ions was measured by the laser-induced fluorescence mapping technique. It was found that the Er^3+^ in the fibers was uniformly distributed along the diameter without an obvious gradient, which preliminarily verified the possibility of cladding the grown fibers. Subsequently, Nie improved the LHPG equipment accuracy and systematically studied the scattering loss of YAG single-crystal fibers. The total loss of the fiber at different wavelengths was obtained as shown in Figure 4a. It was found that the loss of the YAG fiber exceeded the intrinsic loss, and the prospect of growing crystal cladding for YAG fibers was proposed [19].

In 2017, MaxWell et al. proposed a YAG sol–gel filling method and grew YAG crystal fibers with diameters ranging from 30 to 120 µm [20]. As shown in Figure 4b, the YAG fibers were passed through the YAG sol at a certain speed and then a cladding was obtained after drying in air. Subsequent measurements revealed that the fibers with cladding had lower scattering loss. In addition, the YAG crystal fibers were also fabricated by sol–gel combined with centrifugal spinning [23,24]. In 2018, Dubinskii’s team successfully fabricated crystal cladding by the liquid phase epitaxy (LPE) method, and the crystal growth system is shown in Figure 4c [21]. They first achieved pumped laser operation using fully crystalline double-clad Yb^3+^:YAG fibers with a final slope efficiency of 37%. Then, Kim et al. fabricated fiber cladding using the heat/tube collapse method [22]. Their team shaped the glass tubes into fiber insertion devices with different diameters instead of manually inserting the cores, thereby reducing contamination and losses while increasing production. Finally, a double-cladding crystal fiber was successfully fabricated as shown in Figure 4d, but cracks existed between the inner and outer layers due to the mismatch of thermal expansion coefficients. Subsequently, Shaw et al. compared three methods for preparing YAG single-crystal cladding: magnetron sputtering, hydrothermal crystal growth, and liquid phase epitaxial crystal growth [25]. It was found that the cladding prepared using different methods exhibited different degrees of optical and physical uniformity, and the hydrothermal method exhibited the lowest fiber loss.

In summary, LHPG technology exhibits a rapid crystal growth rate, reaching magnitudes on the order of mm/min. Secondly, the melting zone mainly relies on the role of surface tension to replace contact with the crucible, thereby avoiding impurity contamination while getting rid of the limitation of the crucible melting point. However, the equipment employed in LHPG is limited to growing one single-crystal fiber at a time. The growth process is susceptible to interference such as airflow, vibration, or laser power fluctuations, leading to the formation of bubbles and uneven surfaces. Additionally, the technology of fiber cladding is still immature, which restricts the development of this method to a certain extent.

### 2.2. Micro-Pulling down Method

**Figure 5:**
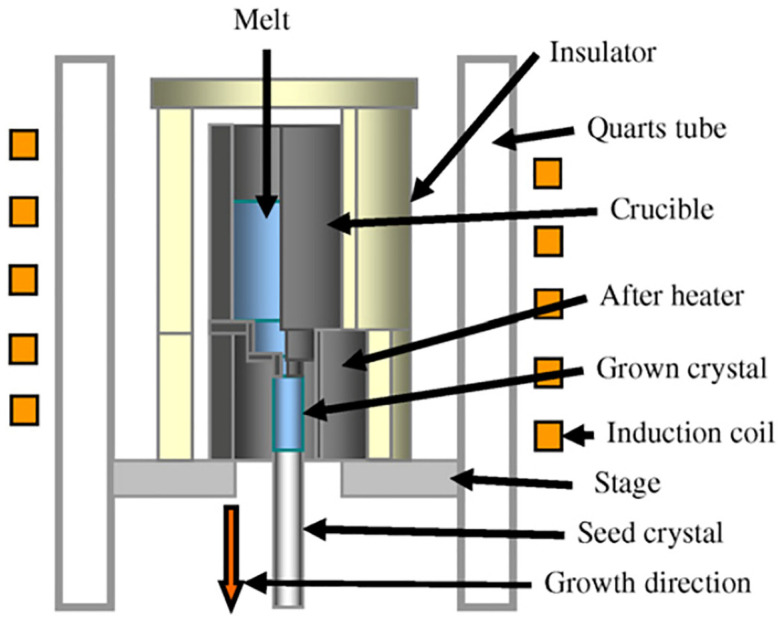
Schematic of the micro-pulling down method [26]. Copyright © 2007 Optical Materials.

Micro-pulling down (μ-PD) is a method for the rapid growth of one-dimensional single crystals. As shown in Figure 5, the melt in the crucible is heated by resistance or radio frequency, and a capillary hole with a diameter ranging from several hundred micrometers to several millimeters is designed at the bottom of the crucible so that the melt can flow out under the effect of gravity. The seed crystals contact with the melt flowing from the capillary holes and are continuously pulled downwards. The process of crystallization occurs when the melt passes through the temperature gradient zone, facilitating the stable growth of single-crystal fibers.

**Figure 6:**
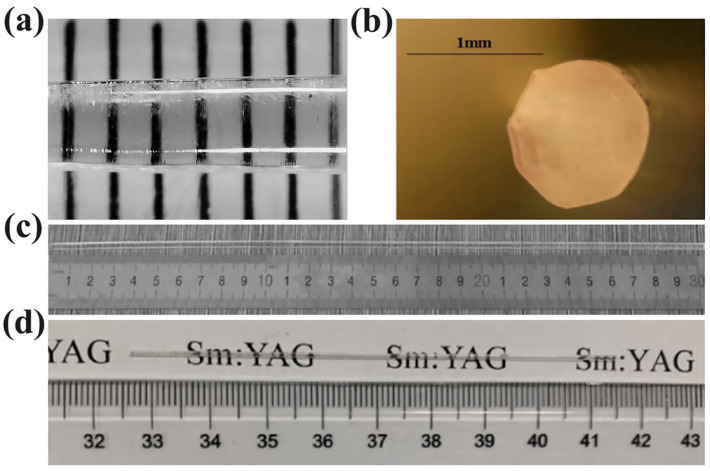
(**a**) Fabrication of Nd^3+^:YAG crystal fibers [27]. Copyright © 1999 Journal of Crystal Growth. (**b**) Schematic cross-section of Nd^3+^:YAG crystal fiber [31]. Copyright © 2007 Optical Materials. (**c**) Nd^3+^:YAG single crystal with 3 mm diameter and 300 mm length [33]. Copyright © 2014 Journal of Synthetic Crystals. (**d**) Sm^3+^:YAG single-crystal optical fibers grown by the micro-pulling down method [34]. Copyright © 2021 Journal of Synthetic Crystals.

The earliest teams fabricating YAG single-crystal fibers by the μ-PD method were in Japan and France. In 1999, Chani at Tohoku University fabricated Nd^3+^:YAG crystals using the micro-pull down method, as shown in Figure 6a, and investigated the segregation and concentration distribution of Nd^3+^ in the crystals [27]. Consequently, his team fabricated Tm^3+^:YAG [28] and Mg/Ce:YAG [29] scintillation crystals and investigated their respective scintillation characteristics. From 2006 to 2011, a series of experiments on the fabrication of YAG single-crystal fibers were conducted by Didierjean et al. at the French Institute of Optics. They fabricated YAG single-crystal fibers with Nd^3+^ doping concentrations of 0.2 at% [30]. The laser-diode end-pumped systems were employed for the first time to conduct laser experiments, resulting in a cw laser power of 10 W at 1064 nm for an incident pump power of 60 W at 808 nm and 360 kW peak power for 12 ns pulses at 1 kHz in the Q-switched regime. Subsequently, they grew crystal fibers with stable diameters ranging from 0.5 to 1.5 mm by an improved μ-PD method [31], as shown in Figure 6b. Through the optimization of parameters including thermal gradient, starting melt composition, and quality control during crystallization, the effective oscillation of the laser in both continuous-wave and Q-switched operation was convincingly demonstrated. In 2011, his team successfully fabricated Nd^3+^:YAG single-crystal fibers with a diameter ranging from 0.3 to 1.0 mm and a doping concentration of 0.2 at% [32]. Moreover, they achieved the first laser operation at 946 nm using Nd^3+^:YAG single-crystal fibers and obtained a maximum output power of 34 W at a pump power of 86 W, which was advanced compared to that reported by Didierjean’s team.

In China, the growth of YAG single-crystal fibers using the μ-PD method was pioneered by Yuan and Tao et al. at Shandong University. In 2014, Yuan’s group grew Nd^3+^:YAG single crystals with a diameter of 3 mm and a length of 300 mm in an argon atmosphere, as shown in Figure 6c. The resulting crystal exhibited excellent transparency, without any internal scattering points [33]. In 2015, the growth process parameters were optimized and a systematic study was conducted on the diameter fluctuation, ion distribution, and optical loss of Nd^3+^:YAG single-crystal fibers. As a result, efficient continuous laser output at 1064 nm was achieved [35]. In 2021, Xu et al. successfully fabricated Sm^3+^:YAG single-crystal fibers for the first time as shown in Figure 6d [34]. After analysis, the absorption coefficient at 405 nm was determined to be 0.24 cm^−1^ and the absorption bandwidth was measured as 1.6 nm. The strongest emission peak occurred at 618 nm, with an emission bandwidth of 3.7 nm, illustrating the potential application of Sm^3+^:YAG single-crystal fibers in red–orange laser output.

In summary, the μ-PD method offers the advantages of reduced raw material consumption and accelerated growth rate [36], thereby significantly reducing the energy cost of crystal growth. Additionally, the most suitable crucible and temperature field can be selected based on different materials’ melting points, viscosity, surface tension, and volatility. However, the fiber fabrication is constrained by the capillary pore size of the crucible and the surface tension of the melt, leading to unstable crystal growth, low fiber quality, and the difficult processing of micron holes. It is evident that the crucible plays a decisive role in the growth process of the μ-PD method, and advancements in crucible development are crucial for future progress in this technique.

### 2.3. Melt-In-Tube Method

**Figure 7:**
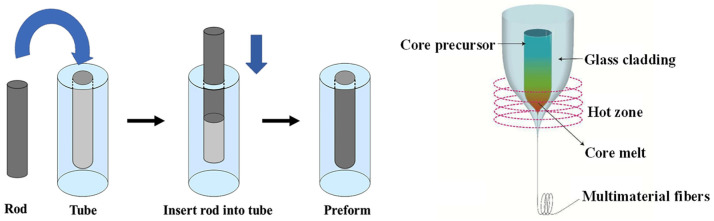
Schematic of preform fabrication (**left**) and drawing process (**right**) [39]. Copyright © 2017 China National Knowledge Infrastructure.

The operational procedures of melt-in-tube (MIT) are similar to the traditional rod-in-tube method [37]. The specific concept of the molten core was initially introduced by Ballato of Clemson University when he observed that the powder was in a molten state during high-temperature drawing processes [38]. As shown in Figure 7, firstly, the core and cladding glass are individually processed to the required size. Secondly, the core rod is inserted into the cladding glass tube to form the fiber preform. Then, the drawing process is conducted using a drawing tower, with precise control of the temperature to ensure core melting while simultaneously maintaining the cladding in a soft state. Finally, the fiber preform is drawn into fibers with the assistance of the traction wheel. The difference between the melt-in-tube and rod-in-tube methods is that the fiber fabricated by the rod-in-tube method can maintain good consistency with the fiber preform in terms of composition and structure. Conversely, the melt-in-tube method creates a large difference in the components between drawn fiber and fiber preform due to the material diffusion and element migration in the high-temperature non-equilibrium state.

The melt-in-tube method can also achieve single-doped or co-doped rare earth by designing the composition and structure of fiber preform, thereby obtaining optical fibers with different characteristics.

#### 2.3.1. Fabrication of Single-Doped Fibers

**Figure 8:**
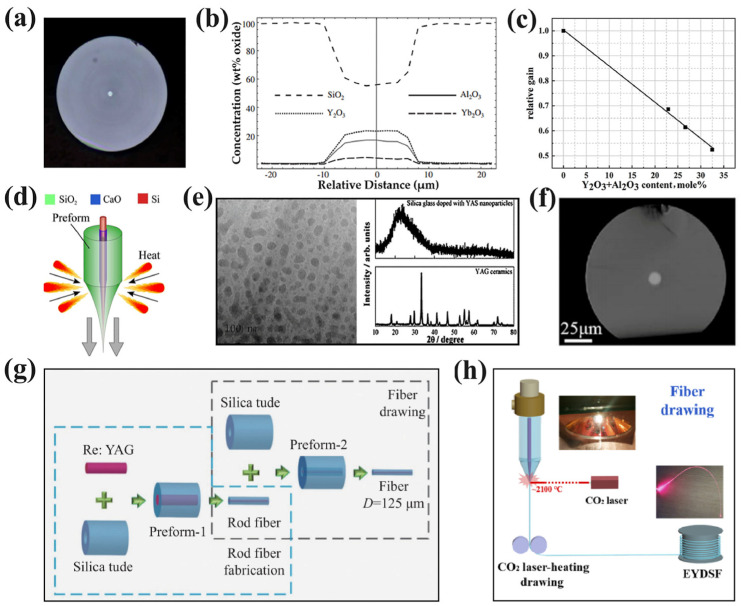
(**a**) Micrograph of an optical fiber with an outer diameter of 125 μm [40]. Copyright © 2009 Journal of Applied Physics. (**b**) Schematic compositional distribution of 10 wt% Yb^3+^:YAG crystal-derived optical fiber [41]. Copyright © 2012 Optical Materials. (**c**) Relative Raman gain (peak) affected by yttrium and alumina content in optical fibers [42]. Copyright © 2013 Electronics Letters. (**d**) Schematic of the tensile structure of optical fiber with a calcium oxide protective layer [43]. Copyright © 2014 Scientific Reports. (**e**) TEM images of the core region and XRD spectra of YAG ceramic and silica glass samples doped with YAS nanoparticles [44]. Copyright © 2016 Optical Materials. (**f**) Electron microscopy images of Tm^3+^:YAG optical fibers [45]. [Reprinted] with permission from ref. [45] © Optics Letters. (**g**) Flow chart of YAS fiber secondary drawing process [46]. Copyright © 2022 Infrared and Laser Engineering. (**h**) Fiber drawing in the Er^3+^:YAG fiber fabrication process [47]. Copyright © 2023 Optics Express.

The initial investigation on YAG-derived optical fibers (YAS) was conducted by Ballato’s team at Clemson University. Their team first combined the melt-in-tube method with YAG crystals to fabricate silica-clad Er^3+^:YAG fibers using undoped and 0.25 wt% and 50 wt% Er^3+^-doped YAG crystals. Furthermore, a comprehensive analysis of the core composition and optical properties was conducted [40]. As shown in Figure 8a, a fiber with a diameter of 125 μm was obtained by the melt-in-tube method, and its core cladding exhibited excellent roundness and concentricity. By analyzing the relationship between the SiO_2_ concentration in the core and the core diameter, it was found that the smaller the core diameter, the more intense the diffusion of Si elements, and the absorption spectra of the YAG fibers indicated that the core was amorphous.

Then, Dragic et al. successfully fabricated Yb^3+^:YAS fibers using the melt-in-tube method for the first time [41]. The compositional distribution of 10 wt% Yb^3+^:YAS fiber is shown in Figure 8b. It can be observed that SiO_2_ constituted the primary component of the fiber core, indicating the diffusion of elements that occurred during the drawing process. Furthermore, spectral measurements and fiber amplifier experiments were conducted on this fiber to analyze its feasibility for use in fiber amplifiers. Considering the lower Brillouin gain coefficient of YAS, an in-depth investigation was conducted by his team: in 2010, Ballato fabricated undoped and 0.05 mol%, 0.25 mol%, and 2 mol% doped Er^3+^:YAG using the melt-in tube-method [48]. The findings revealed that the presence of Y and Al elements in the YAS fiber significantly reduced its photoelastic coefficient compared to pure silica fiber while enhancing the phonon velocity and refractive index of the fiber core. Subsequently, they fabricated Yb^3+^:YAG fibers at 2025 °C [49,50], and the molten YAG eventually solidified into an amorphous core as the fiber cooled. The factors influencing the Brillouin gain in the yttrium–alumina system were analyzed and the gain characteristics of the fiber in the Brillouin zone at 1534 nm were measured. The obtained Brillouin spectrum width was approximately 11 GHz, with a linewidth of about 45 MHz, thereby demonstrating the feasibility of utilizing Yb^3+^:YAS fibers as a gain medium to realize a kW-level narrow linewidth fiber laser. In 2017, Tuggle et al. conducted a comprehensive investigation of Yb^3+^:YAG fibers with a highly stimulated Brillouin scattering threshold [51]. Two kinds of fibers were drawn from the same preform, i.e., fiber 1, with a larger core that dissolved less with SiO_2_ cladding, and fiber 2, with concomitantly less Al_2_O_3_ and Y_2_O_3_. The Brillouin gain spectral widths of fiber 1 and fiber 2 were approximately 200 MHz and 500 MHz, respectively. This discrepancy arose from the smaller core diameter of fiber 1, which was prone to causing the waveguide attenuation of acoustic waves. Furthermore, the relationship between the core diameter, numerical aperture, nonlinear intensity, and Brillouin gain coefficient was further analyzed. In addition to investigating the Brillouin gain of YAS, his team also studied the Raman gain of 5 wt% Yb^3+^:YAG fiber fabricated by the melt-in-tube method [42]. Figure 8c presents a comparative diagram illustrating the relative Raman gain of YAS fibers with varying SiO_2_ contents. It was observed that spontaneous Raman scattering decreased as the SiO_2_ content was reduced, thereby demonstrating the advantage of yttrium aluminum silicate in reducing Raman gain. Considering the serious Si element diffusion in the cladding, as shown in Figure 8d, his group coated CaO as a protective layer within the fiber preform to reduce Si element diffusion under high temperatures and decrease the thermal strain during the drawing process [43,52]. Subsequently, interface modifiers such as CaO, NaO, MgO, and BaO were compared [53]. Among them, the fiber with CaO as the protective layer exhibited superior performance by effectively mitigating thermal mismatch during the drawing process and reducing the viscosity at the boundary between the molten core and cladding. In 2021, his team also investigated the concentration quenching and ion clustering effects of YAS fibers with different Er^3+^ doping concentrations [54]. The experimental results revealed different degrees of Er^3+^ clustering in each fiber, and the ratio of clustered ions was proportional to the silica content and inversely proportional to the concentrations of Al_2_O_3_ and Y_2_O_3_. Consequently, it can be concluded that YAS glass exhibits remarkable capability for accommodating Er^3+^ ions.

In recent years, YAS fibers have also attracted the attention of numerous Chinese research groups. In 2016 at the Shanghai Institute of Optics and Fine Mechanics and Shanghai Institute of Ceramics, researchers successfully fabricated YAS fibers with a diameter of 120 μm using the melt-in-tube method at 1950 °C [44]. Figure 8e illustrates TEM and XRD images of the core region, revealing the presence of nanoparticles in the core region of YAS. Although the XRD analysis revealed the absence of a crystalline structure in the nanoparticles, the YAS nanoparticles inside the core could significantly enhance the nonlinearity of the fiber. The properties of YAS microcrystalline glass have also been investigated in China [55]. Subsequently, in order to mitigate the diffusion of SiO_2_ in the cladding, their team proposed the melt-in-tube method with the post-feeding mode, and 1 at% Nd^3+^:YAG [56] and Yb^3+^:YAG [57] fibers were fabricated using this method. It was found that the SiO_2_ concentration in the Nd^3+^:YAG core reduced from 73.76 wt% to 45.08 wt%, resulting in enhanced output power from 0.45 W to 4 W along with an approximately 30% increase in slope efficiency [56]; similarly, the optimal performance of Yb^3+^:YAG fibers was achieved with a slope efficiency of 42% and an output power of 3.60 W [57]. These findings demonstrate that the utilization of the post-feeding mode effectively reduces the diffusion of Si elements in the fiber. With advancements in raw material fabrication technology and equipment performance, optimized methods and high-performance YAG fibers have been continuously proposed: in 2020 at the South China University of Technology, Qian’s team successfully fabricated a 12 wt% Tm^3+^-doped YAG ceramic-derived fiber [45]. As shown in Figure 8f, the resulting fiber exhibited a core/clad diameter of 10 μm/125 μm and demonstrated a high gain per unit length of 2.7 dB/cm at 1950 nm, representing the highest gain per unit length at 2 µm among similar Tm^3+^:YAG-derived multimaterial fibers at that time. In 2021, Xie et al. at Shandong University fabricated Er^3+^:YAS fibers through a secondary drawing process [58]. A flow chart of the secondary drawing fabrication process is presented in Figure 8g. Firstly, a fiber preform with a YAG crystal was drawn at 1980 °C. Subsequently, the resulting YAS fiber was inserted into a silica tube to form a new preform for the secondary drawing experiment, finally obtaining an Er^3+^:YAG fiber with a diameter of 125 μm. A maximum gain coefficient of 1.46 dB/cm was obtained, which is the highest at the 1.5-μm band reported for YAG crystal-derived all-glass silica fibers. Considering the heating method of the melt-in-tube process also affects diffusion. Wan’s team at Shanghai University used CO_2_ laser heating instead of the traditional graphite furnace heating in 2023 [47]. As shown in Figure 8h, an Er^3+^:YAS fiber was fabricated at 2100 °C, and the CO_2_ laser offered advantages in parameter control and cladding diffusion reduction due to its faster heating rate and smaller high-temperature region. This method effectively mitigated the diffusion of elements under high temperatures, resulting in a gain coefficient of up to 1.74 dB/cm. In the same year, they fabricated Yb^3+^:YAS fibers by the same method and finally obtained a fiber with a core diameter of 9.5 µm and a cladding diameter of 125.2 µm. Remarkably, this fiber exhibited an impressive gain coefficient of up to 6.0 dB/cm at 1030 nm [59].

#### 2.3.2. Fabrication of Co-Doped Fibers

**Figure 9:**
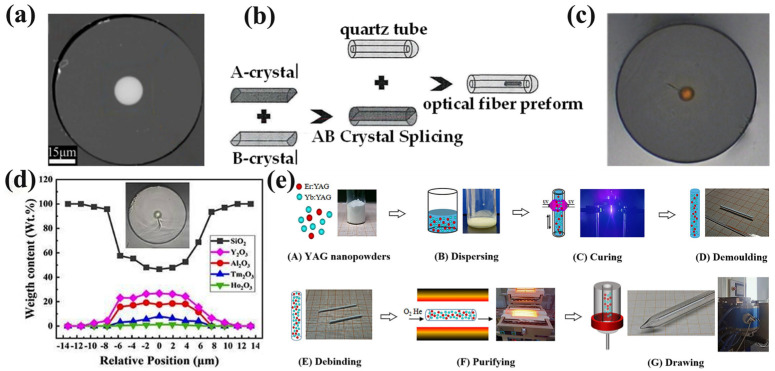
(**a**) Electron microscopic image of a fiber after stretching [60]. [Reprinted] with permission from ref. [60] © Optics Letters. (**b**) Schematic of co-fusion-in-tube method [61]. Copyright © 2021 China National Knowledge Infrastructure. (**c**) Microtomography of Er^3+^/Yb^3+^ co-doped YAG crystal-derived optical fibers [46]. Copyright © 2022 Infrared and Laser Engineering. (**d**) One-dimensional element distribution of the fiber cross-section, where the inset is a microscopic image of the fiber end face [63]. [Reprinted] with permission from ref. [63] © Optics Letters. (**e**) Co-precipitation method for the fabrication of powders and Er^3+^/Yb^3+^ co-doped YAS fabrication flowchart [64]. Copyright © 2022 Journal of the American Ceramic Society.

The fabrication of YAS fibers co-doped with rare earth using the melt-in-tube method has also been a hotspot of research in recent years. In 2019, Tang and his research team inserted Ho^3+^/Cr^3+^/Tm^3+^:YAG crystals into a 15 cm long silica tube to form a fiber preform, drawing at 2000 °C. This process resulted in fibers with an outer diameter of around 125 μm and a core diameter of about 25 μm [60]. As shown in Figure 9a, the core–cladding structure remained intact, without any obvious discontinuity at the interface. Tm^3+^ could be used as an excellent sensitizer to co-dope with Ho^3+^, enabling wavelength extension and achieving a tuning range of approximately 2 μm. The core component is an important factor affecting the performance of fibers; thus, effective adjustment can enhance a fiber’s performance. However, traditional core melting processes face challenges in achieving precise component regulation. To address the above issue, Wei’s team from Shandong University proposed a co-fusion process in the tube [61]. As shown in Figure 9b, crystals were shaped into semi-cylindrical forms and spliced according to desired components. These crystals were then placed within the same silica tube for subsequent fiber drawing experiments. Under high temperatures, the crystals melted and mixed with each other in the tube and finally solidified with the cooling of the fiber. This process enabled the fiber core to possess predetermined components, facilitating the effective regulation of optical fiber performance. An Er^3+^/Yb^3+^ co-doped YAS fiber was fabricated by the co-fusion-in-tube method, as shown in Figure 9c, with a gain coefficient of 2.33 dB/cm and a pump absorption coefficient of 2300 dB/m at 976 nm. Furthermore, the successful realization of DBR single-frequency fiber laser output was achieved using these fibers. Subsequently, an Er^3+^/Yb^3+^:YAG fiber with a diameter of 125 μm was obtained by combining the secondary drawing process and co-fusion-in-tube method at 2000 °C [62]. The maximum absorption coefficient at 976 nm reached 23 dB/cm, which was 8.4 times higher than for the Er^3+^:YAG fiber. Moreover, the measured transmission loss at 1064 nm ranged from 5.3 dB/cm to 7.42 dB/cm, demonstrating excellent gain characteristics and pump absorption efficiency. These results prove the validity and feasibility of component modulation by the co-fusion-in-tube method. In 2023, Wei’s team continued to modulate the composition by splicing 15 at% Tm^3+^:YAG and 2.5 at% Ho^3+^:YAG crystal rods together and fabricated Tm^3+^/Ho^3+^:YAG fibers by co-fusion in the tube at 2000 °C [63]. The one-dimensional distributions of the fiber interfaces are shown in Figure 9d, and the gain coefficient of the resulting gain fiber at 1940 nm was 10.2 dB/cm. A single-frequency fiber laser was researched by using a 1.8 cm long fiber as the gain medium. In addition to the optimization of the melt-in-tube process, some scholars also improved the fabrication process of the raw material powders. Zheng’s team investigated a novel approach for fabricating Er^3+^/Yb^3+^ co-doped YAS fibers using UV-curable nanocomposites [64], as shown in Figure 9e. Firstly, Er^3+^:YAG and Yb^3+^:YAG nanopowders were fabricated by the co-precipitation method, followed by dispersion of the powders in UV-curable resin to form a suspension nanocomposite slurry. Subsequently, the slurry was subjected to curing, debonding, and purification processes to obtain inorganic Er^3+^:YAG/Yb^3+^:YAG rods. Finally, the rod was drawn into Er^3+^/Yb^3+^ co-doped YAS fibers. The absorption coefficients measured at 976 nm and 1530 nm were 17 and 3.2 dB/cm, respectively, and single-frequency laser output at 1.5 μm was achieved.

The melt-in-tube experiment reveals that the core and cladding material of the fiber preform can exhibit complete heterogeneity, whereas traditional fibers must conform to matching factors such as the thermal expansion coefficient, refractive index, softening point, and high-temperature wetting angle. This is attributed to the non-equilibrium state under high temperatures during the drawing process, which promotes a certain degree of compatibility between the core and cladding. Consequently, this feature breaks the limitation and significantly expands the range of compositions for the core material. Furthermore, the rapid cooling of molten cores during high-speed drawing prevents abnormal crystallization and its fabrication is relatively simple and flexible. However, the issue of element diffusion and migration between the core and cladding poses a significant challenge in the fabrication of crystal fibers. Furthermore, the mechanisms of the diffusion phenomenon remain unclear. Therefore, future research should focus on investigating material compatibility and elucidating the mechanism behind element diffusion.

## 3. Applications of YAG-Based Fibers

In recent years, significant advancements have been achieved in active ion-doped fiber laser technology at 1.0 μm, 1.5 μm, and 2.0 μm [65]. Thus far, YAS fibers doped with different active ions (Cr^4+^, Nd^3+^, Yb^3+^, Er^3+^, and Tm^3+^) have been developed for high-power fiber lasers, single-frequency fiber lasers, and mode-locked fiber lasers. Among them, single-frequency fiber lasers exhibit remarkable slope efficiency and output power while offering extensive application prospects in the fields of coherent optical communication, high-precision spectroscopy, gravitational wave detection, laser radars, and hydrophone systems [66,67,68]. On the other hand, as repetition frequency and pulse width continue to improve over time, mode-locked fiber lasers are expected to find widespread utilization in the fields of laser surgery, biomedical imaging, and remote sensing [69,70,71].

**Figure 10:**
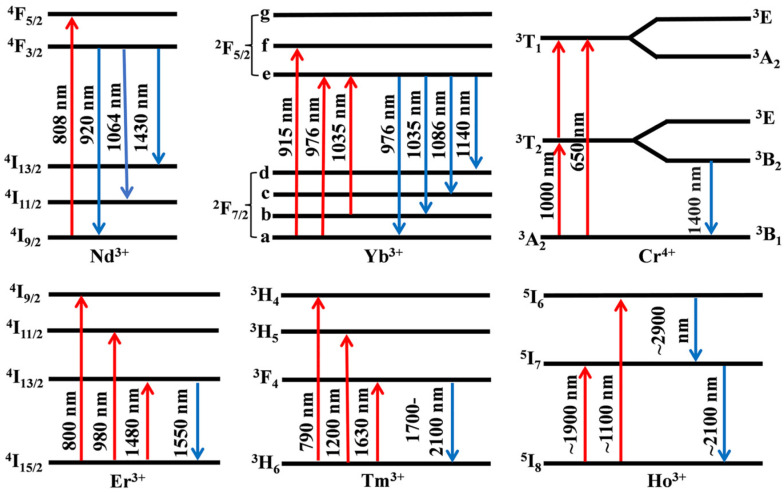
Energy level structure for some representative active ions.

Figure 10 summarizes the energy level structure of representative active ions. The Nd^3+^ ion exhibits a characteristic four-level structure and possesses a low laser threshold, so it is extensively employed in lasers and amplifiers. As depicted in Figure 10, its energy levels primarily consist of ^4^F_3/2_, ^4^F_5/2_, ^4^I_9/2_, ^4^I_11/2_, and ^4^I_13/2_ states. Transitions from the ^4^F_3/2_ state to the ^4^I_9/2_, ^4^I_11/2_, and ^4^I_13/2_ states enable the generation of lasers with varying wavelengths. Notably, the most intense emission peak arises from the transition between the ^4^F_3/2_ and the ^4^I_11/2_ levels at approximately 1064 nm. There are only two electronic states in the 4f layer of the Yb^3+^ ion, and its simple energy level structure eliminates the occurrence of excited state absorption, fluorescence upconversion, and concentration quenching. This characteristic facilitates the realization of high-power laser output. As shown in Figure 10, under external environmental influences, the ^2^F_5/2_ and ^2^F_7/2_ energy levels in the two-energy-level system of Yb^3+^ ions are split into three and four energy levels, respectively. Among these divisions are energy levels denoted as a, b, c, and d that can serve as low-energy particle transition states. Consequently, this enables the generation of excited light between 915 nm and 1035 nm while also producing wide-spectrum emissions ranging from 970 nm to 1140 nm. The Er^3+^ ion, with its rich energy level structure, has found extensive applications in fiber amplifiers. Notably, there are three main absorption processes corresponding to the transitions of ^4^I_15/2_ to ^4^I_13/2_, ^4^I_11/2_, and ^4^I_9/2_, shown in Figure 10. These transitions correspond to pump wavelengths of 800 nm, 980 nm, and 1480 nm. The operating wavelength of 1550 nm aligns with the low-loss band of fiber, and its technology is relatively mature [72,73]. The absorption band of Cr^4+^:YAG crystals is primarily observed at 480 nm, 650 nm, and 1000 nm, with the infrared absorption band at 1000 nm exhibiting a relatively wide range. However, it shows strong excited state absorption due to electron transitions from the ^3^T_2_ excited state to higher energy levels of ^3^T_1_. The emission center wavelength of this laser is within the range of approximately 1400 nm, corresponding to the transition from the ^3^B_2_ state to the ^3^B_1_ state, shown in Figure 10. By employing specific tuning methods, the output wavelength can be adjusted within a range spanning from 1300 to 1600 nm. The unique electron layer structure of Tm^3+^ imparts it with the characteristics of a broad absorption band and a narrow emission spectrum in luminescence, rendering it a common gain medium for achieving laser output at 2.0 μm. Tm^3+^ exhibits three distinct absorption peaks at 790 nm, 1200 nm, and 1630 nm, corresponding to transitions from ^3^H_6_ to ^3^H_4_, ^3^H_5_, and ^3^F_4_, shown in Figure 10. Compared to Tm^3+^, Ho^3+^ exhibits greater gain, a larger excitation cross-section, and a longer fluorescence lifetime at 2 μm. The energy level transitions between ^5^I_7_ to ^5^I_8_ and ^5^I_6_ to ^5^I_7_ correspond to laser oscillations in the wavelength ranges of 1.9–2.15 μm and 2.85–3.05 μm, respectively, shown in Figure 10.

### 3.1. The 1.0 μm Band Fiber Laser

In 2012, Dragic et al. demonstrated the feasibility of utilizing Yb^3+^:YAS fibers as a gain medium, thereby paving the way for the future realization of kW-level narrow linewidth fiber lasers [49]. In 2013 at Charles Fabry’s laboratory [74] and Jena University [75], researchers used single-crystal fibers as an amplified gain medium to inject pulsed light with a repetition frequency ranging from 10 kHz to 10 MHz into a single-crystal fiber for amplification. They successfully achieved a high-power pulse output of 1 mJ at an injection frequency of 10 kHz and slope efficiency reaching 50%. These results provide compelling evidence for the significant potential of Yb^3+^:YAG single-crystal fibers in high-energy amplification, high-power extraction, and high-beam-quality output [76].

**Figure 11:**
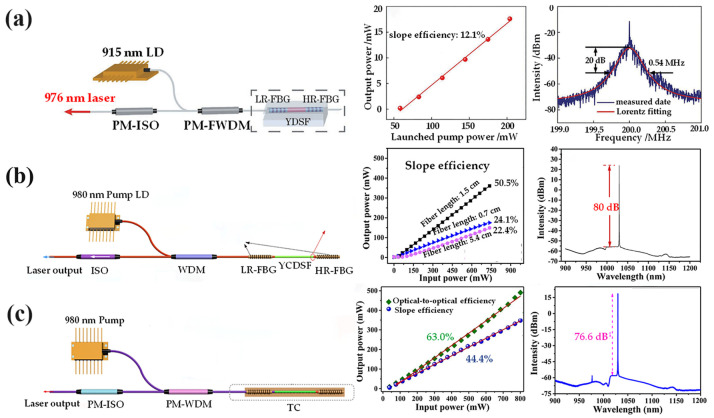
(**a**) Schematic structure of a 976 nm linearly polarized single-frequency Yb^3+^:YAG fiber laser and the linewidth and laser characteristics of the laser [81]. Copyright © 2021 Chinese Journal of Lasers. (**b**) Schematic of all-fiber DBR laser system, the change rule of laser output power with pump power under reverse pumping of different fiber lengths, and the output spectrum of the laser under maximum output power [82]. Copyright © 2020 Optics Express. (**c**) Structural diagram of the DBR linearly polarized single-frequency fiber laser along with the slope efficiency and optical-to-optical conversion efficiency of the laser and the output spectra under maximum output power [59]. Copyright © 2023 Journal of Lightwave Technology.

The Yb^3+^:YAS fiber laser has been in-depth studied by Chinese researchers from the South China University of Technology, Shandong University, and Shanghai University. In 2019, researchers from the South China University of Technology achieved the first single-frequency laser output at 1064 nm based on Yb^3+^:YAG ceramic-derived fibers [77]. However, the slope efficiency was only 3.8% due to the large intracavity loss. In the same year, the group successfully reduced the intracavity loss by decreasing the core diameter of the Yb^3+^:YAS fiber and used the same method to obtain a single-frequency laser at 1064 nm with an improved slope efficiency of up to 18.5% [78]. The Yb^3+^:YAS fiber-based all-fiber integrated-cladding pumped laser, developed by Shandong University, exhibited an output power of 6 W at 1.06 μm and a slope efficiency of 21.7%, with an incident pump power of 28 W [79]. The ring cavity structure facilitated the generation of narrow linewidth lasers, enabling the team to achieve a single-frequency laser output at 1070 nm with a linewidth of less than 4.3 kHz and a slope efficiency of 10.2% [80]. With the advancement of research, YAS fibers below 1 μm have also been successfully fabricated. As shown in Figure 11a, a 976 nm single-frequency laser output with a linewidth of less than 41 kHz and a corresponding slope efficiency of 12.1% was successfully achieved using a DBR linear cavity structure [81].

Researchers at Shanghai University fabricated a Yb^3+^:YAS fiber as a gain medium using the CO_2_ laser-heated melt-in-tube method, resulting in a significant enhancement of the laser performance. In 2020, the team constructed an all-fiber Distributed Bragg Reflectance (DBR) laser using a Yb^3+^:YAS fiber [82]. As shown in Figure 11b, it exhibited a maximum output power of 360 mW, with a pump threshold power of 21 mW and a central wavelength of 1030 nm. Notably, these results surpassed those obtained by any DBR lasers based on Yb^3+^:YAS fibers at that time. Subsequently, they integrated Yb^3+^:YAS into the cavity of a 1030 nm single-frequency DBR fiber laser [83]. Based on this, in order to verify the feasibility of a ring-cavity single-frequency fiber laser as a high-power fiber laser for high-precision fiber sensing, a laser was constructed with Yb^3+^:YAS as the gain medium and Bi^3+^/Er^3+^/Yb^3+^ co-doped fiber as the saturable absorber [84]. The achieved output power at 1030 nm exceeded 100 mW. In 2023, the team fabricated Yb^3+^:YAS fibers with gain coefficients as high as 6.0 dB/cm and built a linearly polarized single-frequency fiber laser with an output power of more than 350 mW, as shown in Figure 11c. This laser demonstrated the highest output power and maximum conversion efficiency among similar single-frequency lasers based on Yb^3+^ ion doping [59]. Additionally, by controlling the polarization state within the cavity, the authors achieved optimal laser performance with a relatively strong noise level below −143.5 dB/Hz and a polarization extinction ratio greater than 21 dB. These findings provide valuable insights for optimizing other rare earth-doped crystal fibers.

The earliest fiber laser at 1.0 μm can be traced back to the Nd^3+^:YAG fiber fabricated by the LHPG method at Bell Laboratories in 1975. Although this fiber was without cladding and did not have a strict fiber structure, it could be applied to a Nd^3+^:YAG laser. With the continuous improvement of the melt-in-tube method, rare earth-doped YAS fibers have attracted significant attention in the field of fiber lasers. Researchers at the University of Southampton pioneered the utilization of YAS fibers in laser applications: Yoo et al. successfully achieved Q-switched pulsed laser operation at 1058 nm using Nd^3+^:YAS [85]. Subsequently, they used the fabricated Nd^3+^:YAS fiber to achieve a continuous laser output at 1058 nm, with a maximum output power of 5.4 W under 808 nm laser cladding pumping conditions, exhibiting a remarkable laser slope efficiency of 52% [86]. Since then, domestic scholars have embarked on the investigation of Nd^3+^:YAS fiber lasers: a team at the South China University of Technology demonstrated a single-frequency laser based on a Nd^3+^:YAS fiber with a gain of 1.8 dB/cm, exhibiting a slope efficiency of 6% at 1064 nm [87]. In 2019, their team also built an all-fiber laser using a Nd^3+^:YAS fiber, achieving more than 50 dB signal-to-noise ratio at 915 nm, indicating the potential for realizing a shorter wavelength with pure blue fiber lasers based on the frequency doubling effect [88]. In 2021 at Shandong University, researchers fabricated a Nd^3+^:YAS fiber using a secondary drawing process and then constructed a DBR single-frequency laser at 1064 nm with a slope efficiency of 1.26%, and the optical signal-to-noise ratio was greater than 50 dB [89]. In conclusion, Nd^3+^- and Yb^3+^-doped YAG fibers can generate laser output at 0.9 μm and 1.0 μm, making them suitable for applications in single-frequency fiber lasers.

### 3.2. The 1.5 μm Band Fiber Laser

**Figure 12:**
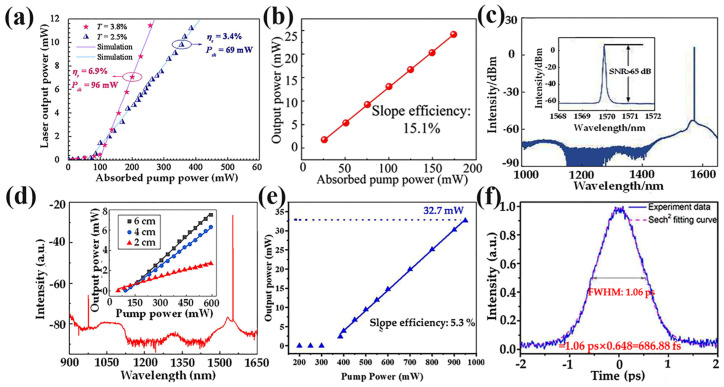
(**a**) Laser characteristics of Cr:YAG fiber lasers [13]. [Reprinted] with permission from ref. [13] © Optics Letters. (**b**) Relationship between output power and absorption pump power of fiber laser [58]. Copyright © 2021 Journal of Lightwave Technology. (**c**) Output spectrum of optical fibers prepared by co-fusion-in-tube method [46]. Copyright © 2022 Infrared and Laser Engineering. (**d**) Relationship between output power and pump power of linear laser with different gain fiber lengths [64]. Copyright © 2022 Journal of the American Ceramic Society. (**e**) Relationship between the output power and the pump power of the ring single-frequency fiber laser based on a Er^3+^:YAS fiber [47]. Copyright © 2023 Optics Express. (**f**) Autocorrelation trace with the sech^2^ fitting curve of the ECDSF-based mode-locked fiber laser at a pump power of 530 mW [91]. Copyright © 2023 Asia Communications and Photonics Conference.

The tunable band of Cr:YAG output lasers ranges from 1300 to 1600 nm, falling within the safe range for human eyes and the low-loss region for optical communication. The first work in fabricating a Cr:YAG crystal laser was conducted by Lo’s team; they successfully generated 2.45 mW of super-wideband ASE using a Cr:YAG fiber and found that as much as 6.5% of Cr ions could exist in a tetravalent coordination state [11]. Subsequently, the team achieved a total gain of up to 10 dB at 1.52 μm with 0.83 W pump power using a double-clad Cr:YAG crystal fiber, which was the first Cr-doped fiber amplifier in the field of fiber communications. The integration of the Cr-doped fiber amplifier with a broadband wavelength division multiplexing (WDM) fiber coupler holds significant potential for applications across the entire range from 1.3–1.6 mm [12]. In order to achieve the realization of a broadband fiber amplifier covering the wavelength range of 1.3~1.6 μm, the group successfully fabricated Cr-doped fibers with a core diameter of 125 μm. The ASE spectrum exhibited a wideband emission spanning from 1.2 to 1.55 μm, which was unattainable in fiber amplifiers at that time [90]. In 2008, the group fabricated a Cr:YAG laser, shown in Figure 12a, achieving a minimum threshold of 69 mW and a maximum slope efficiency of 6.9%. The performance of the Cr:YAG laser could be further improved by optimizing the output coupler transmittance and adjusting the crystal fiber length [13].

In 2021, a 1.55 µm Er^3+^:YAG self-tuned Q-pulsed single-frequency fiber laser was realized for the first time at Shandong University [58]. When the absorbed pump power was 174 mW, the slope efficiency reached 15.1%, as shown in Figure 12b, while exhibiting an output power of 24.2 mW with a phase pulse duration of 78 ns and a repetition frequency of 739 kHz. These findings demonstrate the extensive potential application of Er^3+^-doped YAG fibers in single-frequency fiber lasers at 1.5 μm. Subsequently, they built a DBR short-cavity single-frequency laser and successfully achieved laser output using a Er^3+^/Yb^3+^ co-doped YAG fiber [46], and its output spectrum is shown in Figure 12c. In addition to single-frequency lasers, the team achieved a 1.5 μm mode-locked pulsed laser output at a repetition frequency of 7.45 GHz based on a self-fabricated Er^3+^:YAG fiber. The central wavelength was measured to be 1563.72 nm, while the pulse width and signal-to-noise ratio were determined to be 9.24 ps and greater than 55 dB, respectively [62]. In 2022, Zheng et al. used UV-cured nanocomposites for the first time to fabricate a Er^3+^/Yb^3+^ co-doped YAS fiber [64]. As shown in Figure 12d, its center wavelength was 1552.29 nm and the 3 dB linewidth was 0.094 nm. In 2023 at Shanghai University, an all-fiber continuous-operation ring cavity single-frequency fiber laser with Er^3+^:YAG was first realized [47]. By using only 10 cm fiber as the gain medium, the laser output power was up to 32.7 mW at 1560 nm, as shown in Figure 12e, and a 3 dB linewidth of 660 Hz was estimated. In addition to the single-frequency laser, they also demonstrated an all-fiber mode-locked femtosecond laser employing a Er^3+^:YAG fiber [91]. The mode-locked pulses operated with a duration of 686 fs, as shown in Figure 12f. In summary, Er^3+^ and Er^3+^/Yb^3+^ co-doped YAS fibers are expected to realize high-performance single-frequency fiber lasers with excellent power, linewidth, stability, and noise at 1.5 μm.

### 3.3. The 2.0 μm Band Fiber Laser

In recent years, a team from the South China University of Technology has achieved rich results in the field of YAS fiber lasers at 2.0 μm. The fibers in this range were primarily doped with Tm^3+^, and they systematically studied the effect of Tm^3+^ doping concentration on the optical properties of YAG ceramics. The successful establishment of a Tm^3+^:YAG fiber laser was achieved, demonstrating a remarkable slope efficiency of 12.8% and affirming the viability of utilizing Tm^3+^:YAG fibers as a gain medium for 2.0 μm lasers [92].

**Figure 13:**
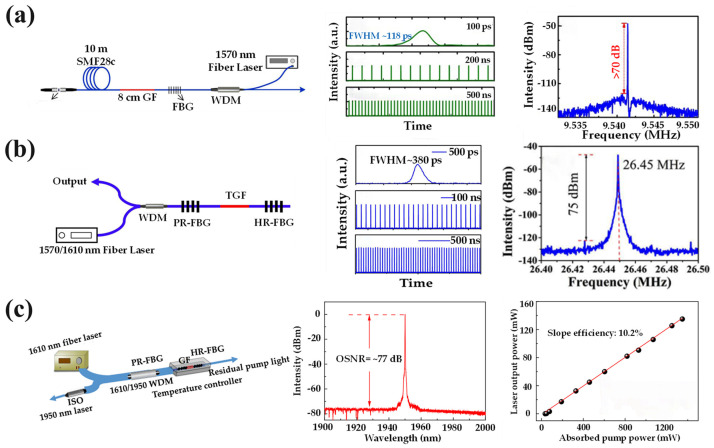
(**a**) Mode-locked fiber laser experimental setup with oscilloscope and RF spectrum recorded in three different time windows [60]. Copyright © 2019 Optics Letters. (**b**) The 1950 nm fiber laser experimental setup with oscilloscope and RF spectrum recorded in three different time windows [45]. Copyright © 2020 Optics Letters. (**c**) Scheme of the experimental setup for a 1950 nm single-frequency DBR fiber laser, the output spectrum of the fiber laser, and the relationship between the laser output power and the absorbed pump power [93]. Copyright © 2022 Chinese Physics B.

In 2019, Tang et al. constructed the first Ho^3+^/Cr^3+^/Tm^3+^:YAG all-fiber-integrated passive mode-locked laser, as shown in Figure 13a [60]. This laser operated at 1.95 μm and exhibited a pulse width of approximately 118 ps, along with a repetition frequency of about 9.5 MHz. Notably, this achievement represents the first realization of mode-locked fiber lasers within YAS fibers. Subsequently, Qian et al. constructed a DBR fiber laser using a 10 cm long Tm^3+^:YAG ceramic-derived fiber, achieving a slope efficiency of 16.5% and a maximum output power of 240 mW. An all-fiber-integrated passive mode-locked laser was realized by pumping with a self-developed fiber laser [45]. As shown in Figure 13b, the experimental setup and test diagram exhibited a working wavelength of 1950 nm, pulse duration of 380 ps, and repetition frequency of 26.45 MHz.

In 2022, Qian also realized a 1.95 µm single-frequency DBR fiber laser based on a Tm^3+^:YAG ceramic-derived fiber [93]. The experimental setup is illustrated in Figure 13c, and an optical signal-to-noise ratio of 77 dB was achieved. The threshold of the single-frequency laser was measured to be 15.4 mW, with a linewidth of 4.5 kHz. The results demonstrate that high-gain Tm^3+^:YAS fibers exhibit promising potential for applications in 2 μm single-frequency fiber lasers. In 2023, Shandong University successfully developed a single-frequency laser utilizing Tm^3+^/Ho^3+^-doped YAG fibers as the gain medium for the first time. The emission wavelength of Tm^3+^/Ho^3+^ was measured at 1940 nm and the achieved output power reached an impressive level of 315 mW, which is the highest output power of a 2 μm single-frequency laser using a crystal-derived fiber [63]. These experiments convincingly demonstrate that YAG fiber holds great potential for applications in single-frequency fiber lasers operating at 2 μm. Furthermore, by optimizing fiber components and laser paths, it is anticipated that laser outputs beyond the 2 μm range can be obtained.

Single-frequency fiber lasers and mode-locked fiber lasers have received more and more attention in recent years, and continuous development has been achieved in many fields. Table 1 and Table 2 present the recent research status of single-frequency and mode-locked lasers using YAG-based optical fibers from different research groups. Breakthroughs in various parameters and wide applications are expected in the future.

## 4. Challenges and Development Trends of YAG-Based Optical Fibers

### 4.1. Challenges in Fabrication and Applications

During the fabrication of YAG-based optical fibers, YAG crystals, ceramics, or powders can be utilized as the filling material for YAG fiber preforms. YAG crystals and ceramics need to be processed into columnar mandrels before being combined with silica tubes; this fabrication process is relatively mature. However, the fabrication cost of a single crystal is high and the ceramics are prone to introducing impurities and pores. Recently, experiments have been conducted to fabricate fiber preforms with YAG powder as a filling material and the feasibility of this method has been proven [94]. This method avoids a series of rod processing while allowing for the flexible design of components and ingredients, thus achieving higher raw material purity levels. Nevertheless, the powder particles tend to yield voids compared to the solid core rod, and the melting of the core is prone to generating more bubbles in the process of drawing under high temperatures. Consequently, experiment stability and operability are relatively low, without a mature process for preparing YAG fibers.

Crystal fibers, as the earliest researched fiber material, have garnered significant attention owing to their diverse physical and chemical properties. However, the development of YAG SCFs has encountered obstacles in recent years. One important factor is the immaturity of SCF cladding fabrication technology. If it is possible to improve the laser performance while reducing the cost of cladding, as well as establish a comprehensive fabrication system, YAG SCFs will make breakthrough progress in the field of optical fibers. In addition to crystal fibers, the YAS multi-component glass fibers fabricated by the melt-in-tube method have become one of the research hotspots in recent years. Scholars have extensively studied YAS fibers doped with various rare earth ions and found that the main component of the fiber is multi-component glass containing Si, Y, and Al elements. The proportion of Si elements even exceeds 50 wt%, replacing the original dominant positions of Y^3+^ and Al^3+^. This leads to the dilution of rare earth ions, resulting in low gain for the fabricated fiber. However, a clear explanation for why Si elements intrude into the core cannot be provided as real-time observation during high-temperature reaction processes is impossible. At this stage, by changing the concentration of rare earth ions in the core, as well as the temperature, rod feeding speed, and other drawing parameters, the core components of the obtained fiber can be analyzed. It has been observed that the Si element exhibits a gradual distribution along the diameter direction within the core, with its lowest content at the center position. Using different drawing parameters will significantly influence the concentration of Si elements in the core, consequently affecting YAS glass’s mesh structure [95,96]. To approximate this gradient of Si element concentration across the radial direction of the fiber cross-section, some researchers proposed a concentric circle structure model resembling tree rings, where each circle represents specific local structural entropy [97]; this could also simulate the removal of inclusions from a thermodynamic perspective [98]. Therefore, the current occurrence of this multicomponent can be attributed to the high-temperature chemical reaction as well as the intensive diffusion and migration of elements. Specifically, the Si elements in the glass cladding under high temperatures diffuse into the core region, which is rich in Y and Al elements, while the continuous increase in internal energy and the expansion of the concentration gradient provide a driving force for diffusion. In addition to elemental diffusion, the extrusion caused by the interfacial stress between the low-viscosity YAS glass and the softened silica cladding is also a possible cause of core multicomponents. In experiments, a tendency towards the symmetric distribution of refractive indices and stresses along the radial direction of YAS fibers was found, which suggests that refractive indices, stress distributions, and the spontaneous elemental migration of silica may interact with each other [99], similar to the dependence between elastic properties and stress, such as the elastic softening of amorphous [100] and crystalline elastic moduli with diffusion-induced stresses [101]. In addition, although an arbitrary matching of core and cladding materials is theoretically possible, if the difference in the thermal expansion coefficients is too large, the fiber will break when subjected to stretching [102], so matching the thermal expansion properties of the cladding and core is required. The melt-in-tube process still needs a lot of exploration and optimization to achieve the fabrication of high-performance functional fibers.

In addition to the fabrication process, there are also many challenges in the application of YAG fibers. The YAG fiber is commonly employed in single-frequency and mode-locked lasers. The gain fiber plays a crucial role in fiber lasers, where the characteristics of laser single-frequency and mode-locking are closely associated with the gain coefficient of YAG fibers. To achieve a narrower linewidth in single-frequency lasers, sufficient frequency separation between adjacent longitudinal modes should be allowed. Since the longitudinal mode spacing is inversely proportional to the cavity length, it becomes necessary to limit the DBR cavity length to several centimeters. Similarly, to obtain high repetition frequency and short pulse duration in mode-locked lasers, reducing the length of the resonator cavity is essential. Therefore, it is necessary to improve the YAG fiber gain coefficient by enhancing the doping concentration of rare earth in the YAG. Currently, due to low concentrations of rare earth ions in fabricated YAG fibers, it is difficult to achieve high gains, which restricts the development of ultra-narrow linewidth single-frequency lasers and high repetition frequency mode-locked lasers.

The high output power characteristics of YAG fiber lasers have also become the main goal of development in recent years. At present, the scattering in the stimulated Brillouin zone and the instability of thermally induced modes in the fiber are the main factors limiting the increase in the output power [103]. Moreover, prolonged high-power output also leads to an increase in the power density of the fiber, which is prone to generating nonlinear effects, thereby leading to mode instability and diminished beam quality. Consequently, addressing how to enhance laser output power while ensuring beam quality remains a focal point and challenge for future research.

Another significant challenge in the application of YAG fibers is the performance degradation caused by losses. Constructing fiber lasers often involves fusing and coupling fibers. Due to the large refractive index difference between YAG and SiO_2_, the fabricated fiber has a relatively large numerical aperture. On the one hand, the fusion with commercial silica fibers results in significant mode mismatch losses, leading to reduced slope efficiency and increased transmission losses in the constructed fiber laser. On the other hand, when fusing with the pigtail fiber, the fusion bonding point is prone to thermal effects and accompanied by the fiber’s own high transmission loss, resulting in serious device heating. The poor thermal stability affects the linewidth and noise of single-frequency lasers to a certain extent.

### 4.2. Development Trend in Fabrication and Applications

During the fabrication of YAG-based optical fibers, the core and cladding are the fundamental components of an optical fiber structure. There are two forms of composite between the core and cladding: one involves growing cladding after crystal growth, which is commonly used in the cladding process after the growth of SCFs. At present, the hydrothermal growth method, the molten salt growth method, and magnetron sputtering are utilized for the growth of cladding. However, their laser characteristics do not meet ideal standards. Liquid phase epitaxy (LPE) technology has been found to grow single-crystal undoped YAG cladding in recent years, enabling the fabrication of all-crystalline fibers. Additionally, some teams have grown SCFs by the LHPG method as the seeds of LPE, resulting in the successful fabrication of double-cladding all-crystalline YAG fibers. Its feasibility as a laser gain medium has been confirmed by testing. Currently, the fabrication process of LHPG crystal fibers is sufficiently mature to support further advancements in all-crystalline fiber development. However, future progress relies on addressing key challenges, such as achieving high-quality and low-loss undoped cladding around doped YAG cores. Therefore, devising a cost-effective and resource-efficient fabrication method while exploring novel fully crystalline fiber production techniques remains pivotal for future development. The other form is the simultaneous growth of crystal and cladding; the melt-in-tube method of preparing fiber has reached a relatively mature stage. On the one hand, future advancements should focus on enhancing the quality of the core material, such as by developing YAG transparent ceramics. The fabrication cost is low relative to the YAG single crystal while minimizing defects like pores and grain boundaries in the core material. Consequently, this reduces impurity introduction and significantly decreases light scattering, thereby mitigating transmission losses in fibers to a certain extent. On the other hand, optimization of the drawing process is necessary, including precise control over drawing temperature: an excessively high temperature will amplify the air pressure’s impact on silica tube deformation and potentially damage the core structure; conversely, excessively low temperatures may result in insufficient softening of the silica tube, leading to the easy breakage of fibers, with poor roundness and concentricity in their cores. Continuous optimization of the process can effectively deal with the uneven distribution of stress in the fiber, cladding that is easy to crack, poor optical properties, and other issues, thus improving the optical performance of fibers.

The crystallization of the core material is a crucial development trend for addressing the core–clad diffusion issue of the melt-in-tube method. To achieve YAG core material crystallization, it is necessary to address the problem of chemical reaction, elemental migration, and interfacial stress at the interface between the core and cladding under high temperatures. At this stage, it is found that the diffusion of Si elements in the cladding primarily occurs after entering the high-temperature region. In order to mitigate this diffusion under elevated temperatures, future approaches may involve adding intermediate barrier layers or implementing the post-feeding method, etc. Furthermore, parameters such as chamber temperature, traction speed, and air pressure in the tube during the fabrication process should be precisely controlled. Furnace chamber temperature affects the rate of elemental diffusion; the traction speed influences the retention time under high temperatures, which subsequently impacts the diffusion time; and the air pressure in the tube may affect the driving force of diffusion. The optimization of the above parameters is expected to achieve the control of the Si element content in the core. In a recent study, it was found that the performance of fibers fabricated by CO_2_ laser heating instead of the traditional graphite furnace heating is more excellent, which is due to the faster heating speed, smaller high-temperature zone, better parameter control capabilities, and reduced cladding diffusion potentialities. The future utilization of CO_2_ laser heating is expected to mitigate diffusion issues to a certain extent. Simultaneously, a clear understanding of the mechanisms governing material matching and element diffusion holds promise for advancing YAG fiber single crystallization technology, enabling the realization of functional crystal advantages and excellent drawing performances of optical glass. This can facilitate the development of composite optical fibers and structure-integrated optical fibers and the integration of microstructured optical fibers, photonic crystal fibers, and orbital angular momentum optical fibers into materials [104].

Yttrium aluminosilicate (YAS) glass fiber has received increasing attention in the field of functional fibers in recent years by virtue of exceptional gain, high thermal conductivity, low nonlinear effects, etc. These factors are primarily attributed to its remarkable rare earth ion solution capability, ensuring outstanding optical or magnetic performance. There are several points to note regarding the future development of YAS glass fiber: 1. The specific formation mechanism of YAS remains unclear and the theoretical model needs further improvement. The formation process and physical properties of the YAS glass network structure can be simulated with the help of molecular dynamics and phase diagram approaches [97,105,106,107], while combining experimental tests to establish the diffusion equation for a multicomponent solid solution [101,108,109]. The composition of YAS fiber is expected to be regulated in the future, which provides a new choice for fabricating controllable gradient refractive index fibers. 2. Through experiments at this stage, it has been found that YAS fiber fabricated by the melt-in-tube method contains YAS quantum dots or nanocrystals in the core in addition to its own glass phase. Although the elemental migration is not conducive to the optical performance of the fiber, the presence of the quantum dots makes the rare earth particles’ luminescence stronger than its own glass coordination field, thereby exhibiting optical properties beyond the glass itself. This finding is of great significance for the development of the YAG quantum dot composite fibers, and the distribution of quantum dots and the effect on the performance of optical fibers should be further investigated in the future. 3. Currently, the doping of YAS fibers is limited to only a few elements. By doping new active ions or improving the doping system of rare earth in the melt-in-tube process, such as through co-doping or the modulation of the ion doping concentration ratio, the fiber laser wavelength range could be expanded. YAS fibers possess the potential to become a widely used matrix material for realizing fiber lasers. In recent years, a co-fusion-in-tube method has been developed and used to realize the variety of rare earth ion co-doping ratios for Er^3+^/Yb^3+^ co-doping, Ce^3+^/Sm^3+^ co-doping, Tm^3+^/Cr^3+^/Ho^3+^ co-doping, etc., which also offers a relatively simple fabrication process. To meet the performance requirements of fiber refractive index, spectrum, bandwidth, etc., flexible design and fabrication of single-mode fibers based on existing technologies is expected.

During the application of YAG-based fibers, single-frequency technology based on YAS functional fibers has mainly focused on low-power single-frequency lasers in recent years, while mode-locked lasers have narrow bandwidths of mode-locked spectra due to intra-cavity grating bandwidth limitations. Future development needs to improve the output power of the YAS fiber laser by increasing the mode field area and shortening the fiber length to inhibit the stimulated Brillouin zone scattering. Additionally, it is necessary to optimize YAS single-frequency and mode-locked fiber laser characteristics by improving matrix glass solubility, such as using the high doping characteristics of YAS fibers to prepare high-gain composite fibers. This will stimulate the application potential in high-power single-frequency lasers with narrow linewidths and mode-locked fiber lasers with higher repetition frequencies and shorter pulse durations.

Nowadays, the application technology of Yb^3+^:YAS-SiO_2_ fibers at 1.0 um is relatively mature, while, at the 1.5 um band, although Er^3+^:YAS fibers have also achieved DBR and ring cavity single-frequency laser output, their efficiency remains relatively low compared to Er^3+^/Yb^3+^ co-doped fibers. Furthermore, the process of Er^3+^/Yb^3+^:YAS fibers is not mature at present, and there is still a large space for development. Tm^3+^:YAS fibers have been verified for application at 2.0 μm and are expected to achieve laser output and application at the band beyond 2.0 μm by using different kinds of laser pumping or co-doping with various rare earth ions in the future.

At this stage, the low efficiency and high loss of YAS fiber lasers are also challenges that need to be solved. For the background loss and transmission loss, it is essential to dry the raw materials in order to minimize hydroxyl and impurity content in fiber preforms and enhance the drawing temperature of the fiber to reduce core viscosity and facilitate pore removal. For the fusion loss caused by mode mismatch, controlling the weld and conducting tests between YAS and silica fibers, along with coating or grinding the end face of the fiber to achieve a more flawless cross-section, can effectively mitigate fusion loss. In addition, the mode mismatch also causes serious thermal effects at the melting point position and destroys the thermal stability of the system; this can be further optimized by adding a strict temperature control system.

Noise is a crucial factor for evaluating the performance of single-frequency lasers. The noise in single-frequency lasers can be categorized into three types: low-frequency noise primarily originates from external environment disturbances, including mechanical and temperature disturbances; mid-frequency noise is mainly associated with relaxation oscillations; and high-frequency noise arises from the shot noise. At present, suppression technologies such as the Michelson fiber interferometer, optical feedback, and optical microcavity are employed to mitigate laser noise in single-frequency lasers. However, these suppression techniques may be susceptible to environmental disturbances and optical feedback mechanisms. In the future, it is necessary to further improve the sensitivity of suppression systems by the use of multi-executor composite suppression, while conducting systematic investigations on suppressing YAS fiber laser noises.

## Figures and Tables

**Table 1:** Research progress of YAG single-frequency lasers.

Core Precursor	Gain (dB·cm^−1^)	Slope Efficiency	Power (mW)	Linewidth (kHz)	OSNR (dB)	Refs
Nd:YAG Crystal (2.5 at.%)	1.49@1064 nm	1.26%	2.58	-	>50	[89]
Nd:YAG Ceramic (5.0 at.%)	1.57@1064 nm	6.0%	20	-	-	[87]
Yb:YAG Crystal (5.66 wt.%)	4.4@1030 nm	34.9%	258	171	79	[83]
Yb:YAG Crystal (15.0 at.%)	6.0@1030 nm	44.4%	350	109	76.6	[59]
Yb:YAG Crystal (4.8 wt.%)	1.7@1064 nm	10.2%	45	<4.3	60	[80]
Er:YAG Crystal (5.0 at.%)	1.46@1550 nm	15.1%	24.2	-	>75	[58]
Er:YAG Crystal (6.5 at.%)	1.74@1560 nm	5.3%	32.7	660	68.1	[47]
Tm:YAG Ceramic (12 wt.%)	2.7@1950 nm	16.5%	240	-	>53	[45]
Tm:YAG Ceramic	2.7@1950 nm	10.2%	135	4.5	77	[93]

**Table 2:** Research progress of YAG mode-locked lasers.

Core Precursor	Gain (dB·cm^−1^)	Repetition Frequency (MHz)	Pulse Duration (ps)	FWHM (nm)	Refs
Ho + Cr +Tm:YAG Crystal (0.3 + 0.3 + 5 at.%)	-	9.5	118	0.09	[60]
Tm:YAG Ceramic (12 wt.%)	2.7@1950 nm	26.45	380	0.38	[45]
Er:YAG Crystal (6.5 at.%)	1.74@1560 nm	14.87	0.686	-	[91]

## Data Availability

Not applicable.
